# Task-shifting to optimize outpatient neurological care in Zambia

**DOI:** 10.1186/s12960-021-00619-7

**Published:** 2021-07-16

**Authors:** Ana C. Villegas, Deanna Saylor, Michelle Kvalsund, Masharip Atadzhanov, Clarence Chiluba, Lorraine Chishimba, Stanley Zimba, Mashina Chomba, Omar K. Siddiqi

**Affiliations:** 1 School of Public Health, Boston University, Boston, MA USA; 2 Department of Internal Medicine, University of Zambia School of Medicine, Lusaka, Zambia; 3 Department of Neurology, Johns Hopkins University School of Medicine, Baltimore, MD USA; 4 International Neurologic & Psychiatric Epidemiology Program, Department of Neurology & Ophthalmology, Michigan State University, East Lansing, MI USA; 5 Global Neurology Program, Department of Neurology, Beth Israel Deaconess Medical Center, Harvard Medical School, 330 Brookline Avenue, Boston, MA 02215 USA; 6 Center for Virology and Vaccine Research, Department of Internal Medicine, Beth Israel Deaconess Medical Center, Harvard Medical School, Boston, MA USA

**Keywords:** Task-shifting, Nurses, Neurology, Sub-Saharan Africa, Zambia

## Abstract

**Objective:**

To investigate opportunities for task shifting to decongest an outpatient neurology clinic in Zambia by describing current patient flow through the clinic and potential nodes for intervention using process mapping.

**Background:**

Zambia has a population of approximately 18 million people with 4 full-time adult neurologists, as of 2018, who all practice at the University Teaching Hospital (UTH), the main tertiary care center in the country. As a result of this provider-to-patient ratio, the outpatient neurology clinic is overcrowded and overbooked. Task-shifting programs have shown to improve efficiency, access and quality of care through the use of less specialized healthcare workers in low- and middle-income countries (LMIC).

**Methods:**

We evaluated patient flow in the UTH neurology outpatient clinic through the development and analysis of a process map. The characteristics of the clinic population between 2014 and 2018 were retrospectively reviewed from the clinic register. Between July and August 2018, we prospectively collected appointment lag times and time each patient spent waiting at various points in the clinic process. We conducted interviews with clinic staff and neurologists to generate a detailed process map of current pathways to care within the clinic. We then devised task-shifting strategies to help reduce patient wait times based on the overview of clinic process mapping and patient demographics.

**Results:**

From 2014 to 2018, there were 4701 outpatients seen in the neurology clinic. The most common neurological diagnoses were epilepsy (39.2%), headache (21.5%) and cerebrovascular disease (16.7%). During prospective data collection, patients waited an average of 57.8 (SD 73.4) days to be seen by a neurologist. The average wait time from arrival in the clinic to departure was 4.0 (SD 2.5) h. The process map and interviews with clinic staff revealed long waiting times due to a paucity of providers. Nurses and clerks represent an influential stakeholder group, but are not actively involved in any activity to reduce wait times. A large proportion of follow-up patients were stable and seen solely to obtain medication refills.

**Conclusions:**

Epilepsy, headache, and stroke make up the largest percentage of outpatient neurological illness in Zambia. Targeting stable patients in these diagnostic categories for a task-shifting intervention may lead to substantially decreased patient wait times. Potential interventions include shifting clinical follow-ups and medication refills to less specialized healthcare workers.

## Background

Neurological disorders represent a costly global public health problem and account for 10.2% of the global disability-adjusted life years (DALYs) and 16.8% of global deaths [1]. With an increasingly older population coupled with limited access to neurological care, the burden of neurological disease in low- and middle-income countries (LMIC) is predicted to continue to increase with a projected 103 million DALYs in 2030 [1].

Previous research has estimated that the average ratio of neurologists to the general population in Africa is on the order of 1:3.4 million, with 11 nations without any neurologists [2]. In comparison, there are between 4 and 13 neurologists per 100,000 people in Europe and the US [3, 4]. Zambia is located in southern Africa and has a high burden of neurological disease with marked delays in accessing neurological care. Its estimated population in 2016 was ~ 18 million [5] with 4 adult neurologists all of whom practice at University Teaching Hospital (UTH), the national referral hospital, in the capital city of Lusaka. As a result of this provider-to-patient ratio, the outpatients:neurologist ratio is extremely high (Table 1).

**Table 1:** Provider-to-patient ratio at the outpatient neurology clinic, 2014–2018

Year	2014	2015	2016	2017	2018	Total
Patients	86	737	1022	1103	1753	4701
Neurologists	1	2	2	2	3	3
Neurologist-to-patient ratio	0.011	0.002	0.002	0.002	0.002	
Zambian population	15,399,788	15,879,361	16,363,458	16,853,599	17,943,895	

This study only collected data from 3 out of the 4 neurologists currently working at UTH

The World Health Organization (WHO) has expressed a need to address under-diagnosis and prompt treatment of non-communicable chronic conditions, recognizing that the model of specialist physician management is not feasible in resource-poor settings [6]. Task-shifting programs have been shown to improve efficiency, access and quality of care through the use of less specialized healthcare workers in specific settings in Africa. This approach is best characterized in HIV/AIDS care but has never been applied to neurological care in LMIC. There needs to be a better understanding of appropriate delegation of tasks by non-specialists in specific populations to successfully implement task-shifting in neurological care. Evidence can help inform strategies to in order to promote better access to neurological care in Africa.

To address this clinical and research gap, we collected essential information on the outpatient neurology clinic at UTH. This study attempted to identify areas to improve efficiency within the UTH outpatient neurology clinic. Based on these findings, we propose an initial frame work for task shifting to aid implementation of neurological care in resource-limited settings.

## Methods

UTH is Zambia’s national referral hospital and is located in the capital city of Lusaka. It is the main teaching hospital for the University of Zambia School of Medicine. The UTH outpatient neurology clinic is staffed by four neurologists who, during the study period, were the only practicing neurologists in the country. This clinic has a neurophysiology laboratory that offers electroencephalograms (EEG) and electromyography/nerve conduction (EMG/NCS) studies. Providers refer patients to the UTH outpatient neurology clinic for specialty evaluation, after hospitalization for a neurological disorder, or for neurophysiology testing.

We employed a mixed quantitative and qualitative approach as there were no national pathways or processes for task-shifting initiatives to guide professionals in neurological care at the time the study was conducted. We elected to use process mapping which incorporates the patient experience to improve the quality or efficiency of clinical management [7]. It is a tool that has shown clinical benefit when applied to healthcare systems [7, 8].

The procedure has been used as a knowledge translation strategy for health systems research in Africa [9]. In Ghana and in South Africa, process mapping has been used as a strategy to combat lung cancer and hypertension [10, 11]. Our approach was novel because process mapping has been used to shape policy change in population health, but has never been used to analyze neurological care. We sought to use process mapping as a quality improvement (QI) tool in order to decrease hurdles associated with implementation of neurological care in resource-limited settings and facilitate further quality improvement efforts.

The team consisted of a Master of Public Health graduate student and two neurologists providing outpatient care in Zambia to review care-delivery processes. We conducted standardized in-person field interviews with clinic staff regarding workflow using a standardized interview guide with question prompts. Data gained from these interviewers were then used to develop process maps. Eligible staff who agreed to participate in the quality improvement research project were English-speaking as is standard in Zambia. We conducted interviews during periods chosen by staff so as not to interfere with clinical duties. During the staff interviews, staff members provided input to process maps to ensure accounting for all tasks involved in clinical care. We then developed a point-by-point checklist of the process from start to finish (Table 2). Based on the information from the process maps, we prospectively collected appointment lag time periods (e.g., time between initial referral to neurology clinic and first consultation in the clinic) and the total time patients spent waiting in the clinic (e.g., time from patient arrival at clinic to being called into the examination room to see a neurologist) between July and August 2018. Neurological complications of HIV infections are common, even in patients on effective antiretroviral therapy. Since task-shifting interventions in Africa have been primarily studied in the context of HIV care provision [12–15], we analyzed these data separately based on patient's HIV status.

**Table 2:** A point-by-point checklist of the patient process from start to finish at the neurology outpatient clinic

Tasks	Completed (Y/N)	Time
Checklist		
Scheduling		
Arrival		
Front desk		
Vital signs		
Examination room		

Patients were not directly interviewed. Instead, they were given a written survey by clinic staff to carry during the visit upon arrival to the registration desk. As patients moved through the clinic, the study team recorded the time at which each stage of the outpatient process (e.g., registration, vitals, visit with the doctor, etc.) began. Once the visit was complete, patients returned the questionnaire to the registration desk. Quantifying these times on the process map, allowed us to quantitatively analyze the time spent on each step of the procedure and identify inefficiencies for potential improvement. We held online team debriefings to discuss the process map results. This allowed all the team members to identify any further problems and provide suggestions.

In addition to the process map and wait times, we reviewed the outpatient log of all patients seen by the three neurologists in the clinic from 2014 to 2018. We placed all patients into categories based on their diagnosis at the time of their visit (Table 3). A number of cases did not have a final diagnosis due to resource limitations. Based on the number of patients in each diagnostic category, we were able to identify high yield neurological conditions for efficiency initiatives.

**Table 3:** Detailed description of the most common disease categories seen at the neurology outpatient clinic

Disease category	Definition
Seizures/epilepsy	Patients who have suffered a seizure or carry the diagnosis of epilepsy
Infectious	Patients whose primary presentation was related to an underlying infection of the nervous system such as cryptococcal meningitis, toxoplasmic encephalitis, or cerebral malaria
Headache/cephalgias	Primary headache disorders such as tension, migraine, and cluster headaches or some related disorder
Neuromuscular	Disorders of the neuromuscular junction, muscle, or motor neuron disease
Movement disorders	Patients with tremor, Parkinson’s disease, ataxia or other related disorders
Neuropathy/radiculopathy	A heterogenous group of peripheral nervous system disorders including diabetic neuropathy, AIDP/CIDP, plexopathies, and radiculopathies
Cerebrovascular disease	Patients with ischemic stroke, hemorrhagic stroke, and/or transient ischemic attacks
Myelopathy	A broad category that included patients with obvious signs of spinal cord dysfunction such as leg weakness, bowel/bladder symptoms, or sensory level without any further details to clarify the diagnosis
Dementia/neurodegenerative	Patients with AIDS–Dementia Complex, Alzheimer’s disease, Huntington’s disease, and/or cerebellar degeneration
Demyelinating	It was restricted to the central nervous system and included cerebral demyelination, acute disseminated encephalomyelitis, and transverse myelitis
Encephalopathy	A cognitive dysfunction of unknown origin whose time course fit an acute/subacute confusional state
Diagnosis unclear	When it was unclear what a patient had, or if they could not be placed into a diagnostic category with great certainty
Other conditions	If patients had a condition that represented < 1% of the study population

This diagnostic category is based on a previous study at UTH [22]

## Results

**Fig. 1:**
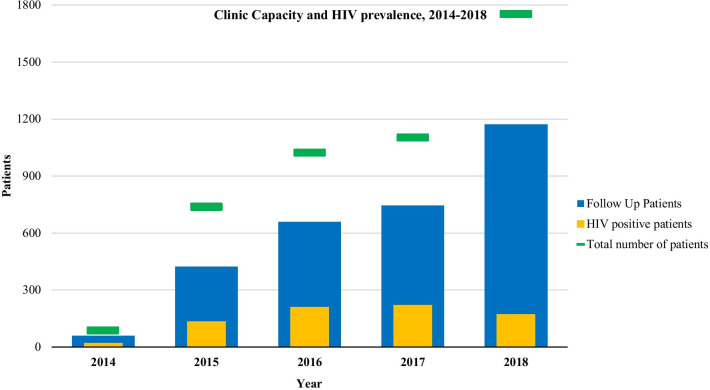
Annual cumulative outpatient visit frequency and prevalence of HIV infection, 2014–2018

**Fig. 2:**
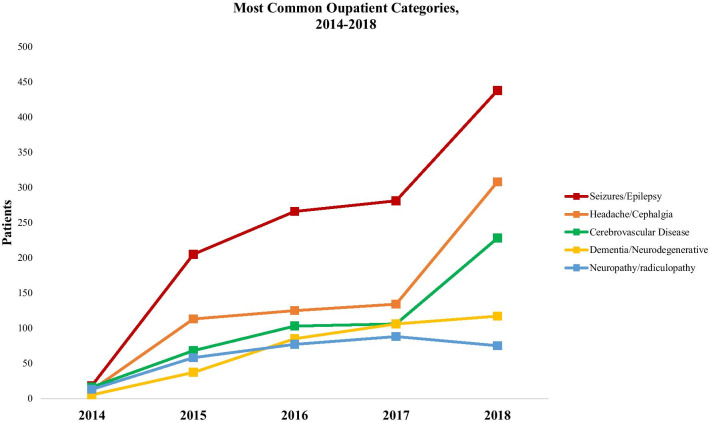
Most common outpatient diagnostic categories, 2014–2018

The annual outpatient visit frequency and prevalence of HIV infection seen at the UTH outpatient neurology clinic over the last 4 years are presented in Fig. 1. From January 2014 to December 2018, the UTH outpatient neurology clinic had 4701 visits, of those 1648 (35.0%) were new patients and 3053 (65.0%) were follow-up patients. The highest volume year was 2018 which included 1753 visits. The median age of patients seen at the outpatient neurology clinic was 42.9 years. A total of 883 (19.0%) were people living with HIV, accounting for 396 (27.5%) of all male patients and 487 (20.5%) of all female patients cared for at the clinic. Figure 2 shows the outpatient neurologic diagnostic categories seen from 2014 to 2018 with epilepsy (39.2%), headache (21.5%) and stroke (16.7%) as the most common.

**Fig. 3:**
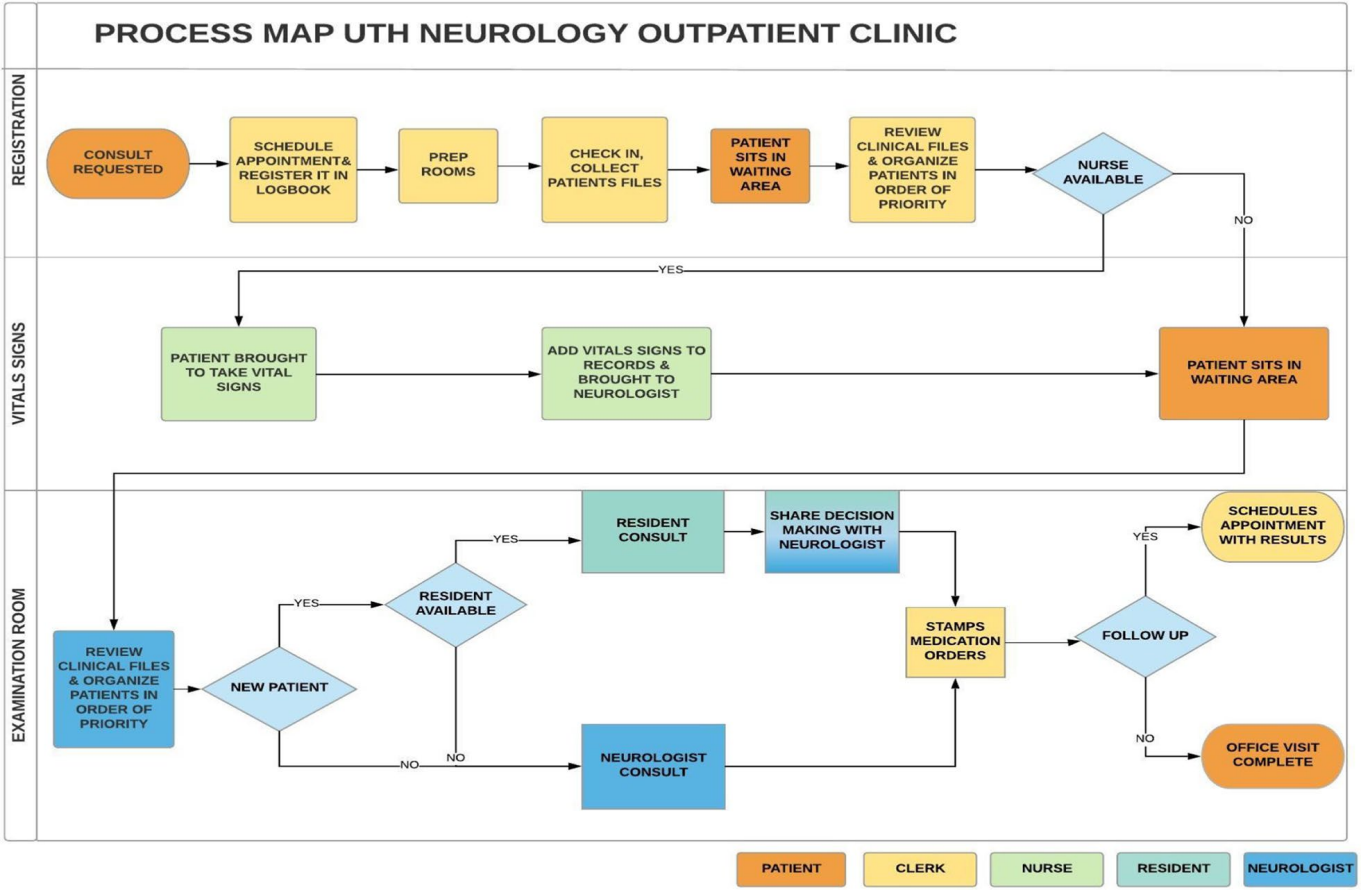
Current process map

Figure 3 provides the finalized process map based on interviews with staff from the UTH outpatient neurology clinic. The process map detailed a challenge for each of the three main steps of the patient flow through the UTH outpatient neurology clinic: registration, recording vital signs, and examination.

The registration desk is the first point of contact for the patients at the UTH outpatient neurology clinic, which runs on a first come, first served basis. Patients start to arrive before its opening to be seen by the team of neurologists as early as possible. Over the course of the study, each physician sees 24.5 patients per week. Of those, only 30% of the patients scheduled an appointment. Once a patient is registered, files are transitioned in the same room to the nurses’ station.

At the nursing station, the nurse takes the patient’s vital signs in the order of arrival. The files are then transferred to the examination rooms. Based on the debriefing sessions with clinic staff and the neurology team, nurses and clerks represent an influential stakeholder group from the registration area. Because of their constant role at the frontline, their practices have a decisive impact. However, often times they were not being effectively utilized. According to the field data, they are not actively involved in any activity to reduce waiting times*.*

Debriefing with clinic staff and neurologists also revealed consensus that long wait times tend to aggravate patients and their families. Examples that were cited include that often stable epilepsy, headache and stroke patients have to wait between 3 and 4 h to obtain a medication refill. They further reported that a large proportion of follow-up patients in these diagnostic categories were stable and largely seen in clinic solely to obtain medication refills. Neurologists also reported that they assume full responsibility for these delays, which leaves them feeling overextended in attempts to see and treat every single patient that presents to clinic that day. As a result, they reported that they commonly see patients on non-clinic days as a strategy to decongest and reduce the workload on clinic days.

Patient-reported appointment lag times and clinic wait times are reported in Table 4**.** On average, patients waited 57.8 (SD 73.4) days to be seen by a neurologist. The average wait time in clinic was 4.0 (SD 2.5) h.

**Table 4:** Patient-reported times waiting and getting into the clinic

Steps	New patient		Follow-up patient	
	Minutes median	IQR	Minutes median	IQR
Appointment lag time (days)	30	39	120	129
Getting to the clinic	40	40	40	37.5
1) Arrival to the clinic (h)	7:15 AM	0:56	7:00 AM	1:16
2) Front desk to vital signs	74	88	67	41.5
3) Hand-off clinical files until in examination room with neurologists	96	198	147	125

*IQR* Interquartile range. 1) Statistics are based on 41 patients over 3 Clinic sessions. 2) All figures are median (IQRS) unless otherwise specified. 3) Not all patients had their vital signs taken (*N* = 9)

## Discussion

**Fig. 4:**
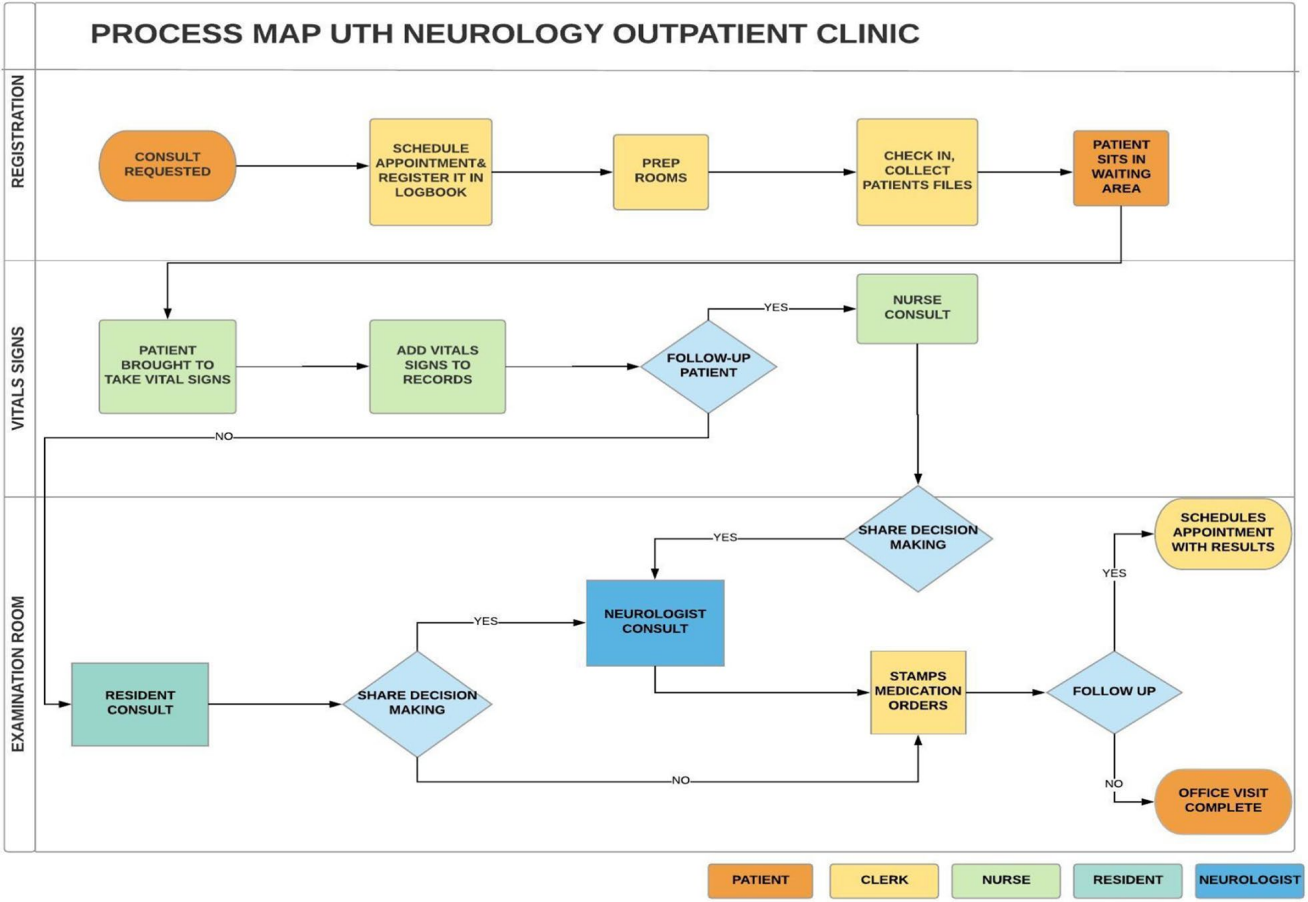
Recommended process map

This study provides valuable information about neurological services at the national referral hospital in Zambia. Consistent with prior studies from the region, seizure, headache and stroke are the most common diagnoses seen at the UTH outpatient neurology clinic [16, 17]. Stable patients in the top three diagnostic categories, requiring only medication refills, were determined to be the optimal target for a task-shifting intervention to reduce patient wait times. Programs targeting medication refills have been shown to improve adherence and decrease physician workload [18–20]. Figure 4 illustrates the recommended approach to the processes around the UTH outpatient neurology clinic, outlining a starting point for effective execution of the task-shifting program in neurology.

Neurology guidelines for upper and middle-income economies recommend individual patient management delivered by neurologists prescribing treatment, supported by imaging and high-technology laboratory work monitoring [21]. Unfortunately, this approach in Zambia and in other LMIC is often not feasible due to lack of neurologists, diagnostic investigations and effective treatments. In fact, the neurological exam remains the most useful diagnostic tool [22]. Our study found that long waiting times for patients to see a neurologist and scarce neurology workforce are adding to the current shortfall. In fact, the UTH outpatient neurology clinic has increased from 1 to 4 neurologists during the study period. In 2018, they experienced the highest patient volume, which likely resulted from the addition of the fourth neurologist. The demand for neurologists, however, continues to exceed supply. Unfortunately, these shortages are likely to be sustained. Thus, guidelines are not translating into clinical practice in these settings because it is not realistic [23]. Alternative strategies to improve access to care for patients with neurological care are urgently needed in LMIC.

Task-shifting to nurses and community health workers may benefit numerous components of the neurology care delivery in LMIC. In Mozambique, training non-physician clinicians in a task-shifted model of antiretroviral therapy rollout for persons living with HIV, compared to physician care, was able to improve health outcomes including viral suppression, toxicity and mortality [24, 25]. Building upon previous findings, we believe common outpatient conditions such as epilepsy, headache and stroke, may benefit from protocols that make it easier for less specialized healthcare workers to manage these conditions.

In this specific context, we recommend shifting clinical follow-ups and refills of chronic medications to nurses, pharmacists and EEG/EMG technicians. These less specialized healthcare workers, however, have had limited training in managing these disorders, so implementation would require an adequate planning and training. A task-sharing model could be adopted first followed by task-shifting as non-physician healthcare workers gained more experience and confidence with their new responsibilities. Ideally, community healthcare workers will be able to determine whether these patients are truly stable and then take accurate treatment decisions. To do so, it is necessary to create a clinical environment that is sufficiently regular and predictable (i.e., only performing clinical evaluations of the same conditions and patient population) because it gives nurses and community health workers the opportunity to learn these conditions through prolonged practice [26]. As the healthcare workers gain more confidence in deciding whether someone is stable or not, the task could be completely shifted to them to identify stable patients without routine discussion with the neurologist.

It is important to note that strengthening training without changes in systems such as clinical operations and procedures, may lead to missing unstable patients, and thus may prove insufficient to improve patient care and/or outcomes [27]. Conversely, we believe that to maximize the accuracy of clinical evaluations made by community healthcare workers, final neurological assessments and decisions should be supported by a checklist of the most common conditions seen at the UTH outpatient neurology clinic. Research has shown that the use of checklists improves clinical outcomes and involves both changes in systems and in the individual and clinical teams’ behavior [28]. The virtues of checklists are well documented in LMIC, where community healthcare workers were able to substantially improve their performances of measure practices with the use of a checklist, as well as improving patient morbidity rates [29].

Based on the Consolidated Framework for Implementation Research (CFIR) [30] creating tension for change is necessary and feasible through better data collection. We encouraged the UTH outpatient neurology clinic to include key performance indicators (kpi): new visit rate, new appointment lag time and no-show rate. These kpis are measurable values that demonstrate how effectively a clinic is achieving the neurology task-shifting goals and overall healthcare objectives. These measures will allow evaluation of this approach, not only for baseline but for ongoing monitoring of the task-shifting program, as well as garner support from leadership. With the creation of urgency, elevating the matter to an organizational aim, there will be an increased awareness among providers, and therefore a stronger culture to improve neurological care using differentiated models of care. Task-shifting can have a relatively quick effect on utilization and on new appointment lag time by increasing the capacity of the services. Hence, there will be a need for shorter evaluation cycles of this neurology task-shifting model, that focuses on process optimization, avoiding radical workflow and operational changes. Task-shifting interventions in HIV populations in Africa may be used as a benchmark for comparing efficiency in a potential task-shifting intervention in neurology in similar countries.

This study has several limitations. The current study only considered the view of patients and providers in a national referral hospital and not populations in more rural settings in Zambia. Since our study is limited in its geographic reach, the findings may therefore be biased towards this population’s experiences and preferences. However, process mapping is a method that can be adapted and used to identify gaps in other outpatient neurology clinics in LMICs, which in turn can help those clinics to tailor the use of differentiated models of care to meet their identified needs.

In addition, our data brought to light other issues in the clinic such as the low appointment bookings rate, which raises the question of what communication barriers patients are experiencing when trying to book appointments in advance. Unfortunately this information was not collected as it was out of the scope of the project. These issues warrant further consideration and may pose opportunities to better communicate with patients by reinforcing the need to schedule appointments in advance or allowing patients to schedule appointments with the use of mobile apps and other technologies. Finally, this study focuses on a clinic in which fully trained neurologists are available to evaluate new patients and support community healthcare workers participating in the task-shifting models. Our suggestions would likely not apply to less-resourced settings in which no neurologists were present.

In Zambia, as happens in other LMICs, the treatment gap of neurological disorders is due in part to a small or absent neurology workforce leading to long waiting times to meet the growing demand for their services. Prospective solutions to reduce lag times may include task-shifting neurological care to nurses and community health workers. This approach will require training, so less specialized healthcare workers can develop the skills needed to identify and make the most appropriate referrals to neurologists and to resume management of stable neurology patients.

## Data Availability

Raw interview data are not publicly available due to them containing information that could compromise participants privacy. Derived datasets used and/or analyzed during the current study are available from the corresponding author on reasonable request.

## Abbreviations

UTH: The University Teaching Hospital
LMIC: Low- and middle-income countries
DALYs: The global disability-adjusted life years
NCS: Nerve conduction
EEG: Electroencephalograms
EMG: Electromyography studies
